# The Incremental Prognostic Value of Baseline ^18^F-FDG PET/CT Imaging in Angioimmunoblastic T-Cell Lymphoma

**DOI:** 10.1155/2020/4502489

**Published:** 2020-06-06

**Authors:** Hui Wang, Wenjing Yu, Tao Wu, Yangyang Xue, Dan Zhang, Huiqin Xu

**Affiliations:** Department of Nuclear Medicine, The First Affiliated Hospital of Anhui Medical University, Hefei, 230022, China

## Abstract

**Methods:**

From January 2010 to October 2019, a total of 23 patients who pathologically confirmed to have AITL were retrospectively analyzed. All patients underwent whole-body ^18^F-FDG PET/CT scan before chemotherapy. The ^18^F-FDG PET/CT features, clinical data, laboratory indicators, Ki67 labeling index, and survival status were collected and analyzed.

**Results:**

The median follow-up was 22 months. The expected 1-, 2-, and 3-year survival rate was 72.2%, 49.6%, and 42.5%, respectively. The median overall survival (OS) was 23 months (95% confidence interval (CI): 8.459~37.541). AITL is prone to extranodal infiltration, in addition to nodal infiltration (6 patients had nodal infiltration alone, and 17 patients had both nodal and extranodal infiltration). The SUV_max_ of nodal lesions were higher than that for the extranodal lesions (10.43 ± 4.45, 6.64 ± 3.51, *F* = 2.78, *t* = 4.39, *P* < 0.01). On multivariate survival analysis, the Eastern Cooperative Oncology Group (ECOG) and SUV_max_ of extranodal lesions were independent predictors of OS.

**Conclusion:**

Baseline ^18^F-FDG PET/CT results and SUV_max_ of extranodal lesions showed an incremental prognostic value in addition to clinical prognostic factors.

## 1. Introduction

Angioimmunoblastic T-cell lymphoma (AITL) is a rare subtype of peripheral T-cell lymphoma diagnosed according to the World Health Organization (WHO) criteria presented in 2001. AITL accounts for approximately 1-2% of non-Hodgkin's lymphoma and 15-20% of peripheral T-cell lymphoma (PTCL) with unique clinical, imaging, and pathological features [1]. AITL has the characteristics of rapid disease progression and poor prognosis. The early symptoms of the disease are not obvious, and patients are in the clinical stage III/IV at the time of diagnosis. The clinical course of AITL is complex and the treatment response is different. Humeniuk et al. [2] reported a case of AITL that went into spontaneous remission, an uncommon occurrence. Besides, the majority of AITL patients showed an aggressive course and dismal outcome with current therapies [1, 3]. Studies showed that ^18^F-fluorodeoxyglucose positron emission tomography/computed tomography (^18^F-FDG PET/CT) is a rapidly evolving hybrid imaging technique in evaluation of infection and cancer; however, it has been rarely applied for predicting outcome of AITL. Therefore, additional information on the predictive value of the PET could be of great significance, especially in association with AITL patients' OS. The ^18^F-FDG PET/CT features, clinical data, laboratory indicators, Ki-67 labeling index, and survival status of 23 patients with AITL were retrospectively analyzed in the present study.

## 2. Study Subjects and Methods

### 2.1. Study Subjects

A total of 23 AITL patients who underwent pretreatment ^18^F-FDG PET/CT from January 2010 to October 2019 were enrolled in the present study. Inclusion criteria were set as follows: (1) histopathologically confirmed as AITL; (2) all other nodal or extranodal lesions were proven to be AITL infiltrations based on image examination and clinical follow-up; and (3) availability of imaging and nonimaging data for staging. Patients with secondary malignant tumor were excluded. This study was approved by the Ethics Committee of The First Affiliated Hospital Of Anhui Medical University. Informed consent was waived because of the nature of this retrospective study.

### 2.2. Observational Indicators

Sex, age, first symptoms, history of autoimmune disease, lactate dehydrogenase (LDH), albumin, C-reactive protein (CRP), beta 2-microglobulin (*β*_2_-MG), bone marrow biopsy, international prognostic index (IPI), and Ki-67 labeling index were collected and analyzed. Patients' basic characteristics are summarized in Table 1.

Ann Arbor staging system was used, and the physical condition was scored as 1~5 according to the Eastern Cooperative Oncology Group (ECOG). The final follow-up deadline was February 2020, and the medical records available in hospital or telephone follow-up were checked. The overall survival (OS) was defined as time from diagnosis to date of death due to any cause or date of last follow-up contact for patients who were alive.

### 2.3. PET/CT Scanning Protocol

All patients underwent whole-body ^18^F-FDG PET/CT scans using a Siemens Biograph TruePoint PET/CT scanner (Siemens AG, Munich, Germany). After 6 h of fasting, PET/CT scan was carried out at 50~60 min after intravenous administration of 3.70~5.55 MBq/kg of ^18^F-FDG, with radiochemical purity >95% (Nanjing Jiangyuan Andike Positron Research and Development Co., Ltd., Nanjing, Jiangsu, China). Blood glucose level was monitored before scanning to ensure that the mentioned level was less than 11.0 mmol/l. The CT parameters were as follows: 120 kV, 80 mA, and PET acquisition was performed at 1 min per bed position for body and 2.5 min per bed position for head. All PET/CT images were interpreted by two experienced nuclear physicians retrospectively using a standard workstation (Syngo MMWP; Siemens AG, Munich, Germany). Focal or diffuse FDG uptake above background in a location mismatched with normal anatomy or physiology was interpreted as abnormal and indicative of a lymphoma lesion. The maximum standardized uptake values (SUV_max_) were determined on PET scans.

### 2.4. Statistical Analysis

Herein, SPSS 17.0 software (IBM, Armonk, NY, USA) was used to carry out statistical analysis. Continuous variables were presented as mean ± standard variation (SD) or median (range) as appropriate. Qualitative variables were expressed as number (%). The optimal diagnostic critical values of SUV_max_ and Ki67 labeling index were obtained by using receiver operating characteristic (ROC) curve (Table 2). OS was determined by Kaplan-Meier analysis, and differences among the groups were analyzed by the log-rank test. Cox proportional hazards model was used for multivariate survival analysis. A *P* < 0.05 was considered as statistically significant.

## 3. Results

### 3.1. Patients' Outcome and Related Clinical Data

The median age of the 23 patients was 65 years old (range, 29~79 years old), and male : female ratio was 1.56 : 1. The most common presenting symptom was superficial mass (13 cases, 56.52%), followed by fever, cough, expectoration, rash, sore throat, abdominal distention, and abdominal pain. Besides, 6 (26.09%) cases had autoimmune diseases, including rheumatoid arthritis, psoriatic arthritis, ankylosing spondylitis, and urticarial vasculitis.

Ann Arbor staging system showed that 20 cases were at stage III~IV. The ECOG score >1 was found in 5 cases. Additionally, 12 cases had an international prognostic index (IPI) score at the range of 3~5. Moreover, elevated LDH and CRP levels were noted in 18 and 23 cases, respectively; the increased *β*_2_-MG level was found in 18 cases; the low level of albumin was detected in 17 cases; Ki − 67 labeling index ≥ 45% was found in 15 cases; and 8 cases had serous cavity effusion as well.

### 3.2. ^18^F-FDG PET/CT Imaging Findings

**Figure 1:**
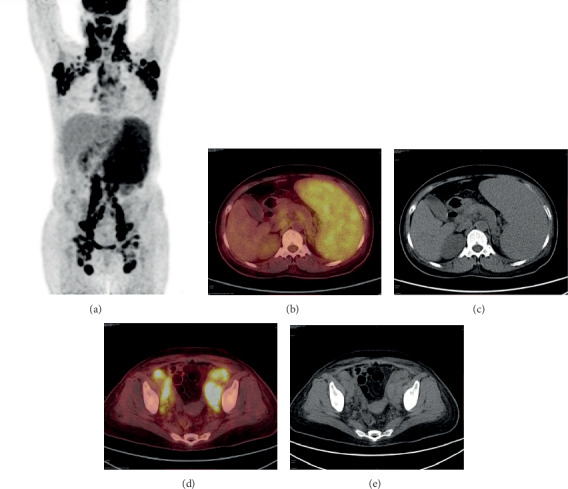
Image captured from a 55-year-old female AITL patient. PET/CT images: (a) body sites; (b, c) an axial PET and CT image show large splenic infiltration (SUV_max_ = 4.6), no change in density in CT; (d, e) illustrate infiltrated lymph nodes in bilateral iliac region (SUV_max_ = 10.9).

**Figure 2:**
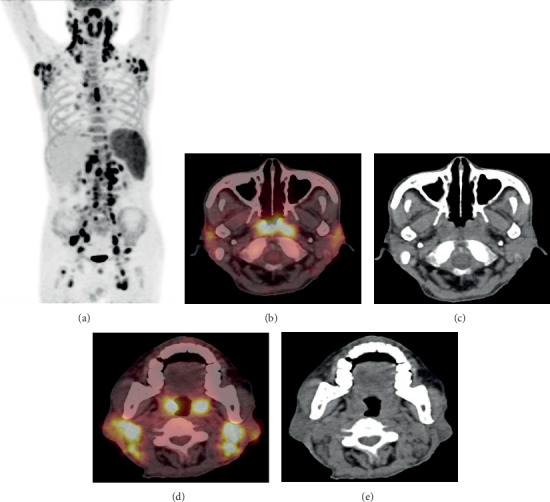
Image captured from a 65-year-old male AITL patient. PET/CT images: (a) whole-body maximum intensity projection (MIP) image displays infiltration in multiple body sites; (b, c) an axial PET and CT image show infiltration in pharynx nasalis with high ^18^F-FDG uptake (SUV_max_ = 11.3); (d, e) illustrate infiltrated bilateral tonsils (SUV_max_ = 12.5).

The imaging findings of ^18^F-FDG PET/CT unveiled that all 23 AITL patients had nodal infiltration (6 patients had nodal infiltration alone, and 17 patients had both nodal and extranodal infiltration). No patient had extranodal infiltration alone. Lymphoma lesions showed positive uptake of ^18^F-FDG (Figures 1 and 2). The distribution of ^18^F-FDG PET/CT in AITL patients with nodal infiltration was as follows: (i) multiple lymph nodes with scattered distribution were observed in 21 patients and (ii) localized distribution: only 2 patients were affected by diaphragmatic ipsilateral lymph nodes, including abdominopelvic cavity, retroperitoneum, and iliac vascular region.

The SUV_max_ of the lymph node infiltration lesions and extranodal lesions was 10.43 ± 4.45 and 6.64 ± 3.51, respectively. The SUV_max_ of nodal lesions was higher than that of extranodal lesions (*F* = 2.78, *t* = 4.39, *P* < 0.01).

The most common extranodal organs or sites were spleen (14 cases), nasopharynx (9 cases), tonsil (7 cases), bone (4 cases), gut (1 case), lung (2 cases), pleura (4 cases), and skin and muscle (1 case). Invasion of spleen was detected in 14 cases, of whom, increased diffuse FDG uptake and no change in density in CT scan were detected. Additionally, no change in bone destruction or bone marrow cavity was found in patients with bone invasion. Notably, two cases with focal lesions on PET/CT scan were confirmed to have false-negative results of bone marrow biopsy.

### 3.3. Survival and Prognosis Analysis

**Figure 3:**
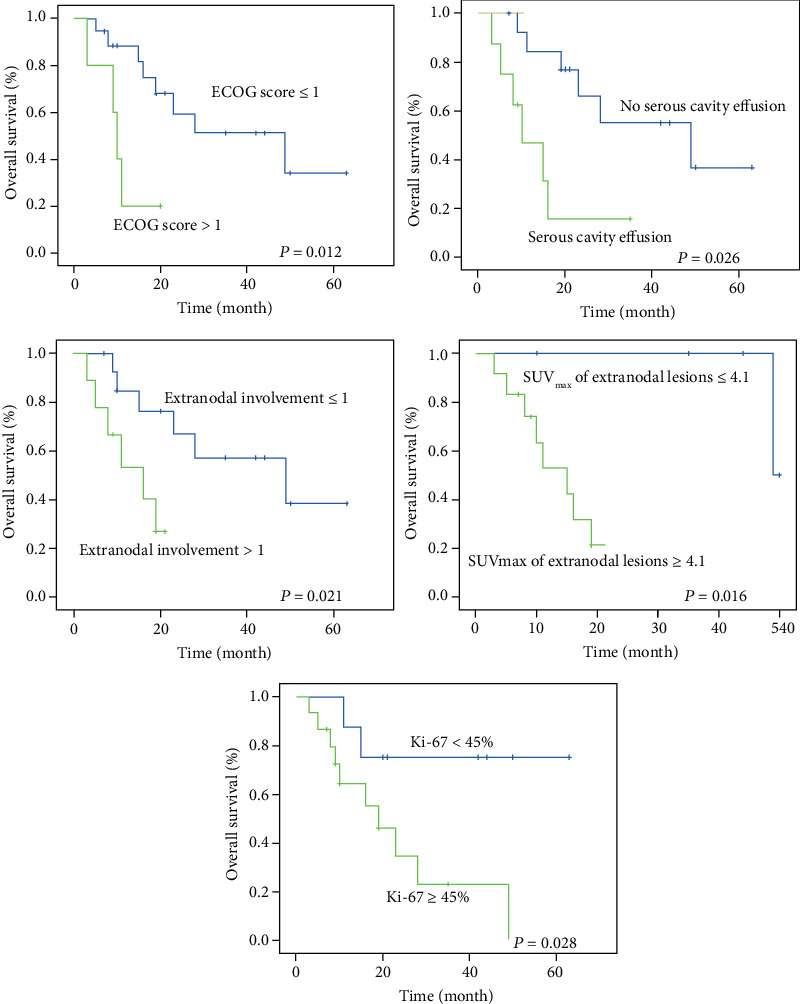
Kaplan-Meier estimate of overall survival by ECOG score, serous cavity effusion, Ki-67 labeling index, extranodal involvement, and the SUV_max_ of extranodal lesions. The optimal cut-off values were obtained by using ROC curve analysis.

The expected 1-, 2-, and 3-year survival rate was 72.2%, 49.6%, and 42.5%, respectively. The median OS was 23 months (95% CI: 8.459~37.541). The log-rank analysis showed that ECOG score > 1, serous cavity effusion, Ki − 67 labeling index ≥ 45%, extranodal involvement > 1, and the SUV_max_ of extranodal lesions ≥ 4.1 were adverse prognostic factors of AITL (*P* < 0.05) (Figure 3). On multivariate survival analysis, ECOG and SUV_max_ of extranodal lesions were independent predictors of OS (Table 3).

## 4. Discussion

AITL is a rare subtype of peripheral T-cell lymphoma with rapid disease progression and poor prognosis. In the present study, the expected 1-, 2-, and 3-year survival rate was 72.2%, 49.6%, and 42.5%, respectively, and the median OS was 23 months (95% CI: 8.459~37.541). Similarly in the previous studies, the 5-year survival rate of AITL patients was lower than 40% [4, 5]. Xu and Liu [6] conducted a large population-based study using the Surveillance, Epidemiology, and End Results (SEER) program (1973-2010) to determine the temporal survival trends and prognostic factors for AITL patients. The results revealed that there was no survival improvement in AITL patients over the past two decades.

^18^F-FDG-PET/CT plays a pivotal role in the assessment of malignant lymphoma. However, a limited number of scholars concentrated on its application in the prognosis of AITL. The present study disclosed that AITL is prone to extranodal infiltration, in addition to nodal infiltration. AITL never caused extranodal infiltration alone in the current research. In addition, patients with extranodal infiltration typically exhibited infiltration in multiple organs, and the most common organs to develop infiltration were the spleen and nasopharynx, followed by the tonsil, bone, lung, pleura, skin, and muscle. This feature is similar to the published reports of PET in the management of AITL patients [1]. High cell turnover and high ^18^F-FDG avidity were noted in the majority of AITL patients demonstrated in the literature [7, 8]. In our study, the SUV_max_ of the lymph node infiltration lesions and extranodal lesions were 10.43 ± 4.45 and 6.64 ± 3.51, respectively. Shao et al. [9] reported the SUV_max_ of lesions with lymph node infiltration and extranodal organ infiltration in AITL patients were 5.4-25.1 (median, 9.7) and 1.5-12.5 (median, 5.5), respectively. This could explain the high aggressiveness of AITL.

^18^F-FDG-PET/CT has been recommended for prognostic analysis for malignant lymphoma [10]. In the current research, baseline ^18^F-FDG-PET/CT results, SUV_max_ of extranodal lesions, and ECOG were independent predictors of OS on multivariate survival analysis. So far, SUV_max_ was the most widely studied parameter with promising results. Gallicchio et al. [11] demonstrated that a baseline SUV_max_ > 13 predicts a poor outcome in patients with diffuse large B-cell lymphoma. In addition to SUV_max_, Deauville score scale and semiquantitative and quantitative PET/CT parameters including metabolic tumor value (MTV) and total lesion glycolysis (TLG) have been demonstrated good results in prediction of response and prognosis in lymphomas. Deauville Criteria (DC) which is based on the application of a five-point scale using the mediastinum and liver activity as the reference standard has been demonstrated good results in prediction of response and prognosis in lymphomas at interim and end-of-treatment PET/CT [12, 13]. Fallanca et al. [14] reported that a score of at least 4 (DC4) showed a high diagnostic accuracy and predicted value for Hodgkin lymphoma and non-Hodgkin lymphoma. More recently, MTV and TLG have been demonstrated prognostic role in survival outcome of many lymphomas at baseline PET/CT [15, 16]. Various MTV delineation methods have been reported such as SUV ≥ 2.5, SUV ≥ 41%, and SUV ≥ mean liver uptake (PERCIST) [17, 18]. However, MTV and TLG seemed to be more suitable for solid tumors than diffuse hypermetabolic organs like the spleen and bone. We chose SUV_max_ as the evaluation index for AITL due to the characteristic of extranodal infiltration.

In the current research, the main demiological (gender, sex), B symptom, Ann Arbor stage, IPI score, laboratory indicators (LDH, Albumin, *β*2-MG), serous cavity effusion, histopathplogical (ki67 labeling index), SUV_max_ of infiltrated lymph nodes, and extranodal involvement >1 site were not associated with OS. Nevertheless, the prognostic factors of lymphoma are controversial. Tokunaga et al. [19] elucidated the clinicopathological characteristics and prognosis of AITL patients in Japan and found that patients' age >60 years old elevated white blood cell (WBC) and IgA levels; the presence of anemia and thrombocytopenia and extranodal involvement at >1 site were significant prognostic factors for OS. Albano et al. [20] revealed that the end of treatment ^18^F-FDG PET/CT significantly associated with PFS, not with OS in mantle cell lymphoma. Similar to our results, Zhou et al. [21] reported that baseline SUV_max_ was independent predictors of OS in peripheral T-cell lymphomas (PTCL). Baseline ^18^F-FDG PET/CT seems to be advantageous in prognosis of AITL. The current research revealed that the metabolic information of extranodal lesions should be concerned by researchers.

The limitation of the current study was the number of patients who enrolled in this retrospective analysis. The incidence of AITL is rare; therefore, further multicenter researches need to be conducted.

## 5. Conclusions

AITL is highly aggressive with poor prognosis. The current research revealed that the metabolic information of extranodal lesions should be concerned by researchers. Baseline ^18^F-FDG PET/CT results and SUV_max_ of extranodal lesions showed an incremental prognostic value in addition to clinical prognostic factors.

## Figures and Tables

**Table 1:** Patients' outcome and related clinical data.

	Case *n* (%)	Median OS (month)	*P*
Gender			0.291
Male	14 (60.87%)	49	
Female	9 (39.13%)	15	
Age (years)			0.844
≥60	16 (69.57%)	28	
<60	7 (30.43%)	23	
B symptom			0.056
Yes	13 (56.52%)	19	
No	10 (43.48%)	49	
ECOG score			0.012^∗^
≤1	18 (78.26%)	49	
>1	5 (21.74%)	10	
Ann Arbor stage			0.071
I~II	3 (13.04%)	42	
III~IV	20 (86.96%)	15.5	
IPI score			0.400
0~2	11 (47.83%)	49	
3~5	12 (52.17%)	23	
LDH (U/L)			0.579
Abnormal (>250)	18 (78.26%)	23	
Normal (≤250)	5 (21.74%)	19	
Albumin (g/L)			0.218
Abnormal (<40)	17 (73.91%)	19	
Normal (40-55)	6 (26.09%)	49	
*β*2-MG(0.9~2.3 mg/L)			0.650
Abnormal (>2.3)	18 (78.26%)	23	
Normal	5 (21.74%)	42	
Serous cavity effusion			0.026^∗^
Yes	8 (34.78%)	10	
No	15 (65.22%)	49	
Ki-67			0.028^∗^
≥45%	15 (65.22%)	19	
<45%	8 (34.78%)	28	
SUV_max_ of infiltrated lymph nodes			0.202
≥7.85	18 (78.26%)	19	
<7.85	5 (21.74%)	23	
SUV_max_ of extranodal lesions			0.016^∗^
≥4.1	12 (70.59%)	15	
<4.1	5 (29.41%)	49	
Extranodal involvement			0.021^∗^
≤1	14 (60.87%)	49	
>1	9 (39.13%)	16	

LDH: lactate dehydrogenase; CRP: C-reactive protein; *β*_2_-MG: beta 2-microglobulin; IPI: international prognostic index; ECOG: Eastern Cooperative Oncology Group; OS: overall survival; SUV_max_: the maximum standardized uptake values. Compared within groups: ^∗^*P* < 0.05.

**Table 2:** Optimal thresholds for predicting patient mortality.

	Ki67 (%)	SUV_max_ of infiltrated lymph nodes	SUV_max_ of extranodal lesions
Optimal threshold	45%	7.85	4.1
Sensitivity (%)	83.3%	100%	88.9%
Specificity (%)	54.5%	37.5%	75.0%
Area under the ROC curve	0.659	0.618	0.806

**Table 3:** Multivariate analysis for survivals.

	OS		
	HR	95% CI	*P*
ECOG	7.089	1.238~40.604	0.028∗
Serous cavity effusion	3.403	0.864~13.399	0.080
Extranodal involvement	0.729	0.088~6.027	0.770
SUV_max_ of extranodal lesions	16.319	1.416~188.082	0.025∗
Ki-67	2.820	0.281~28.329	0.378

ECOG: Eastern Cooperative Oncology Group; OS: overall survival; SUV_max_: the maximum standardized uptake values. Compared within groups: ^∗^*P* < 0.05.

## Data Availability

The data used to support the finding of this study are available from the corresponding author upon request.
